# Functional Expression of TRP Ion Channels in Endometrial Stromal Cells of Endometriosis Patients

**DOI:** 10.3390/ijms19092467

**Published:** 2018-08-21

**Authors:** Eleonora Persoons, Aurélie Hennes, Katrien De Clercq, Rita Van Bree, Goede Vriens, Dorien F. O, Daniëlle Peterse, Arne Vanhie, Christel Meuleman, Thomas Voets, Carla Tomassetti, Joris Vriens

**Affiliations:** 1 Laboratory of Endometrium, Endometriosis & Reproductive Medicine, Department of Development and Regeneration, KU Leuven, Herestraat 49 box 611, 3000 Leuven, Belgium; eleonora.persoons@kuleuven.vib.be (E.P.); aurelie.hennes@kuleuven.vib.be (A.H.); katrien.declercq@kuleuven.vib.be (K.D.C.); rita.vanbree@kuleuven.be (R.V.B.); goede.vriens@kuleuven.be (G.V.); dorien.o@kuleuven.be (D.F.O); danielle.peterse@kuleuven.be (D.P.); arne.vanhie@uzleuven.be (A.V.); christel.meuleman@uzleuven.be (C.M.); carla.tomassetti@uzleuven.be (C.T.); 2 Laboratory of Ion Channel Research, Department of Cellular and Molecular Medicine, KU Leuven, VIB Center for Brain & Disease Research, Herestraat 49 box 802, 3000 Leuven, Belgium; thomas.voets@kuleuven.vib.be; 3 Leuven University Fertility Center, University Hospitals Leuven, Herestraat 49, 3000 Leuven, Belgium

**Keywords:** endometriosis, TRP channels, endometrial stromal cells, eutopic and ectopic endometrium

## Abstract

Endometriosis is a common gynecological disease that is characterized by the presence of functional endometrial-like lesions in the abdominal cavity. Aside from epithelial cells, these lesions consist of stromal cells that have the capacity to migrate, adhere, proliferate, and induce neuro- and lymphangiogenesis, which allows them to survive at ectopic locations. However, the exact underlying mechanisms that regulate these changes are yet to be elucidated. The common ground of these processes, however, is the second messenger, calcium. In this regard, members of the superfamily of transient receptor potential (TRP) ion channels, which are known to be calcium-permeable and expressed in the endometrium, have emerged as key regulators. Here, we assessed the molecular and functional expression of TRP channels in stromal cells isolated from the eutopic endometrium of endometriosis patients and controls. Using RT-qPCR, high mRNA levels of TRPV2, TRPV4, TRPM4, TRPM7, TRPC1, TRPC3, TRPC4, and TRPC6 were observed in the whole endometrium throughout the menstrual cycle. Additionally, and in line with previous reports of control patients, TRPV2, TRPV4, TRPC1/4, and TRPC6 were present in human endometrial stromal cells (hESC) from endometriosis patients both at the molecular and functional level. Moreover, proliferation and migration assays illustrated that these parameters were not affected in stromal cells from endometriosis patients. Furthermore, comparison between eutopic and ectopic endometrial samples revealed that the RNA expression pattern of TRP channels did not differ significantly. Collectively, although a functional expression of specific ion channels in hESCs was found, their expression did not correlate with endometriosis.

## 1. Introduction

**Figure 1:**
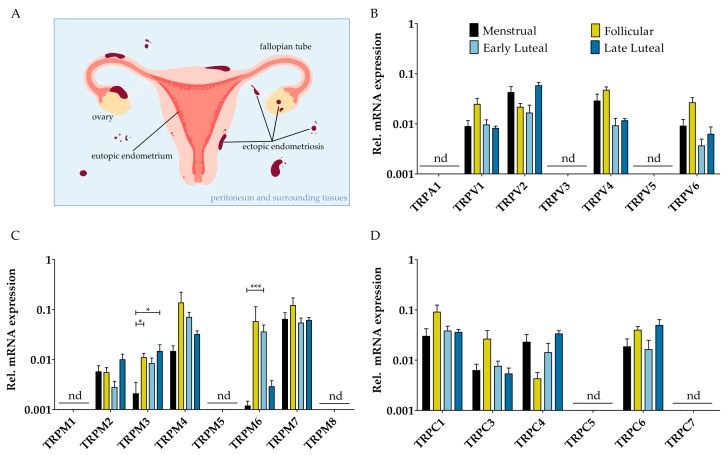
**mRNA expression of TRP channels in eutopic endometrium**. (**A**) Retrograde menstruation is defined as the regression of eutopic endometrium through the fallopian tubes into the abdominal cavity. In patients with endometriosis, this may ultimately result in ectopic lesions on the peritoneum and surrounding tissues, as indicated in a cartoon; (**B**–**D**) Messenger RNA levels of members of the TRPV, TRPM, and TRPC subfamily, respectively, relatively quantified to the geometric mean of housekeeping genes ACTB, GAPDH, HPRT, PGK-1, and TBP. Biopsies were obtained from endometriosis patients diagnosed with grade II [37] throughout the menstrual cycle, including the menstrual (*n* = 5), follicular (*n* = 6), the early luteal (*n* = 4), and the late luteal phase (*n* = 3). nd: not detectable. Data are presented as mean + SEM. Statistically significant changes in mRNA expression were assessed using the Two-Way ANOVA test with Bonferroni correction, * *p* < 0.05, *** *p* < 0.001.

Endometriosis is a gynecological disease, characterized by the presence of functional, endometrial-like lesions located outside the uterine cavity. This chronic illness burdens 6–10% of women who are of reproductive-age [1]. Typically, endometriosis is presented with symptoms such as infertility and/or recurrent abdominal pain, which have a crippling effect on patients’ lives and an immense impact on their healthcare services [2]. In spite of extensive research, the etiology of the disease remains an enigma. Literature suggests several possible etiological theories for endometriosis; however, no single theory can adequately explain all aspects of this disease. Currently, the most commonly accepted theory is the retrograde menstruation of Sampson, i.e., that lesions occur due to the shedding of the eutopic endometrial lining via the fallopian tubes into the abdominal cavity (Figure 1A) [3]. As this is a natural process which occurs in 90% of reproductive women [4], endometriosis lesions are assumed to acquire additional capacities, such as migration, adhesion, proliferation, and neuroangiogenesis, in order for them to establish and flourish in the abdominal cavity [5].

The endometrium comprises primarily of two different cell types: epithelial and stromal cells. The former can be divided further into luminal and glandular epithelial cells which line the lumen of the uterus and the uterine glands, respectively. Together with the endometrial stem/progenitor cells [6], the stromal cells are the driving force behind the regenerative capacity of the endometrium. They have a mesenchymal background, as stromal cells are vimentin positive [7], bestowing them an inherently migratory and proliferative character. During the follicular phase of the menstrual cycle, the stromal cells are subjected to estrogen, leading to cell proliferation and, subsequently, to the thickening of the endometrium. The exposure to progesterone during the luteal phase will result in the differentiation of the estrogen-primed stromal cells into decidual cells. By undergoing this differentiation process, decidual cells will provide an optimal environment for a possible embryo to be implanted [8]. Several research projects have shown that on several accounts the eutopic endometrium of endometriosis patients is different to that of controls [9]. The most striking difference, is the gain of P450 aromatase expression and activity in the stromal cells of endometriosis patients, which allows for local estrogen production [10,11,12]. Furthermore, a deficiency of 17β-hydroxysteroid dehydrogenase type II in these cells, which facilitates the inactivation of estrogen into estrone [13], gives the disease an estrogen-dependent character. The endometriotic lesions—presumed to originate from the endometrium—are also comprised of glandular epithelium and stromal cells. Moreover, the ectopic lesions appear to respond in a similar way to cyclic changes of steroid hormones, such as the endometrium [14,15,16]. However, immunohistochemistry and cDNA microarray studies have shown that the ectopic lesions do not completely resemble their eutopic counterparts [17,18]. They demonstrated an aberrant expression of adhesion molecules [19], anti-apoptotic proteins [20], as well as angiogenic factors, such as the vascular endothelial growth factor [21].

Migration, adhesion, proliferation, and neuroangiogenesis are intricate processes wherein calcium is described as an important regulator [22,23]. Therefore, ion channels are intriguing candidates to regulate these processes, as the activation of ion channels can modulate the intracellular calcium concentrations. The superfamily of transient receptor potential (TRP) channels presents itself as a good candidate to regulate such processes as migration, adhesion, proliferation, and neuroangiogenesis [24,25]. The mammalian TRP-superfamily consists of six subfamilies, based on sequence homology: ankyrin-rich (TRPA1), vanilloid (TRPV1-6), canonical (TRPC1-7), melastatin-like (TRPM1-8), polycystin (TRPP2/3/5), and mucolipin (TRPML1-3) [26]. They can be activated by a variety of stimuli, and are widely distributed throughout the entire body. In endometrial biopsies, TRP channel expression has been shown to fluctuate throughout the menstrual cycle [7]. Furthermore, high mRNA levels for TRPV2, TRPV4, TRPC1/4, TRPC6, TRPM4, and TRPM7, and the functional expression of TRPV2, TRPV4, TRPC6, and TRPM7 was previously illustrated by our group in primary human endometrial stromal cells (hESC) [7]. Interestingly, for some of these stromal TRP channels, their involvement in processes like cell migration (TRPC1/C4 and TRPV2) [27,28], cell adhesion (TRPC4) [29], and cell proliferation (TRPV2, TRPM4, TRPM7) [30,31] has been shown. In addition, Mg^2+^ is involved in essentially every step of cell proliferation, with cancerous cell growth representing the most detrimental effect of deregulated proliferation. Interestingly, TRPM7 represents a major Mg^2+^-uptake mechanism in mammalian cells [32,33] and has been implicated as a regulator of cell proliferation [34], inducing cell cycle arrest if blocked. This is based on channel function in Mg^2+^ transport, as cell growth can be restored by Mg^2+^ supplementation [35,36]. Therefore, the characterization of TRP channel expression between (i) the eutopic tissue of endometriosis and controls, and (ii) the eutopic and ectopic endometrium of endometriosis patients would be an interesting feature towards the understanding of the pathogenesis of endometriosis. To this end, mRNA expression studies, and functional Ca^2+^-microfluorimetry and proliferation/migration assays were performed on human biopsies and the primary cell cultures of both endometriosis patients and controls, to investigate the contribution of TRP channels in stromal cells towards the development of endometriosis.

## 2. Results

### 2.1. mRNA Expression Profile of TRP Channels in Endometrial Biospies and Primary hESC of Endometriosis Patients

Quantitative RT-PCR (RT-qPCR) showed that TRPV1, TRPV2, TRPV4, TRPV6, TRPM4, TRPM6, TRPM7, TRPC1, TRPC3, TRPC4, and TRPC6 were expressed well above the detection limit (Cq < 30) throughout the menstrual cycle in whole endometrial biopsies of endometriosis patients. The mRNA expression level of TRPV3, TRPV5, TRPM2, and TRPM3 was around the detection limit (30 < Cq < 35) of the RT-qPCR analysis, whereas TRPA1, TRPM1, TRPM5, TRPM8, TRPC5, and TRPC7 were not detected (Cq ≥ 35) (Figure 1B–D). The expression levels of these channels was relatively constant throughout the four different menstrual phases, as no significant differences could be found. The expression of TRPM3 and TRPM6, however, did fluctuate significantly between the menstrual-follicular-late luteal phase and the menstrual-early luteal phase, respectively. When comparing these results with expression data of controls [7], no significant changes in the fold change were observed (Figure S1).

**Figure 2:**
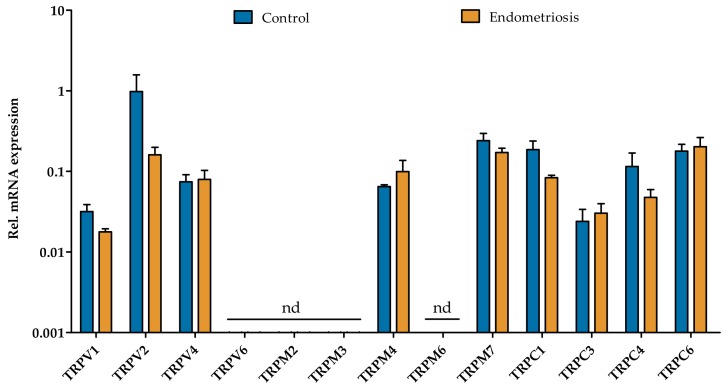
**Quantitative RT-PCR of TRP channels in human endometrial stromal cells (hESC) derived from controls and endometriosis patients.** Primary hESC were cultured from freshly isolated endometrium obtained during the luteal phase of controls (*n* = 3) and grade II endometriosis patients [37] (*n* = 4). mRNA levels were quantified to the geometric mean of housekeeping genes HPRT1 and PGK-1. TRPV6, TRPM2, TRPM3, and TRPM6 were around (30 < Cq < 35) or below the detection limit (Cq > 35). nd: not detectable. Data are presented as mean + SEM. Statistically significant changes in mRNA expression were assessed using the Two-way ANOVA statistical test with Bonferroni correction.

As stromal cells—the most abundant cell type in endometriotic lesions—have adhesive, migratory, and proliferative capacities, human endometrial stromal cells were isolated from both controls and endometriosis patients during the luteal phase. The TRP channel expression pattern was further investigated using RT-qPCR, although only the TRP channels that were present in the whole biopsies were further investigated. mRNA expression of TRPV1, TRPV2, TRPV4, TRPM4, TRPM7, TRPC1, TRPC3, TRPC4, and TRPC6 could be observed in hESC of endometriosis patients (Figure 2). The expression of TRPV6 and TRPM6 was around the detection limit (30 < Cq < 35), while TRPM2 and TRPM3 mRNA levels were below the detection limits (Cq ≥ 35). Furthermore, these levels were not significantly different from the TRP channel expression in hESC from the controls (Figure 2), which is illustrated by a Spearman correlation coefficient of 0.94 (Figure S2). In addition, these results are in line with the expression pattern of the TRP genes in whole endometrial biopsies of endometriosis patients during the luteal phase (Figure 1B–D). Overall, these results showed no difference in the level of TRP channel expression between control and endometriosis patients in endometrial biopsies and in primary cultures of stromal cells of the eutopic endometrium.

### 2.2. Functional Expression of TRP Channels in Endometriosis-Derived hESC

In order to assess the functionality of the TRP channels expressed in hESC of endometriosis patients, Ca^2+^ microfluorimetry was performed, using specific pharmacologic agents. The protocol consisted of the stimulation of hESC with a specific TRP channel agonist, followed by a wash-out period. All primary cells that responded to the application of the positive control stimulus ionomycin (2 µM) at the end of the protocol were used for further analysis.

**Figure 3:**
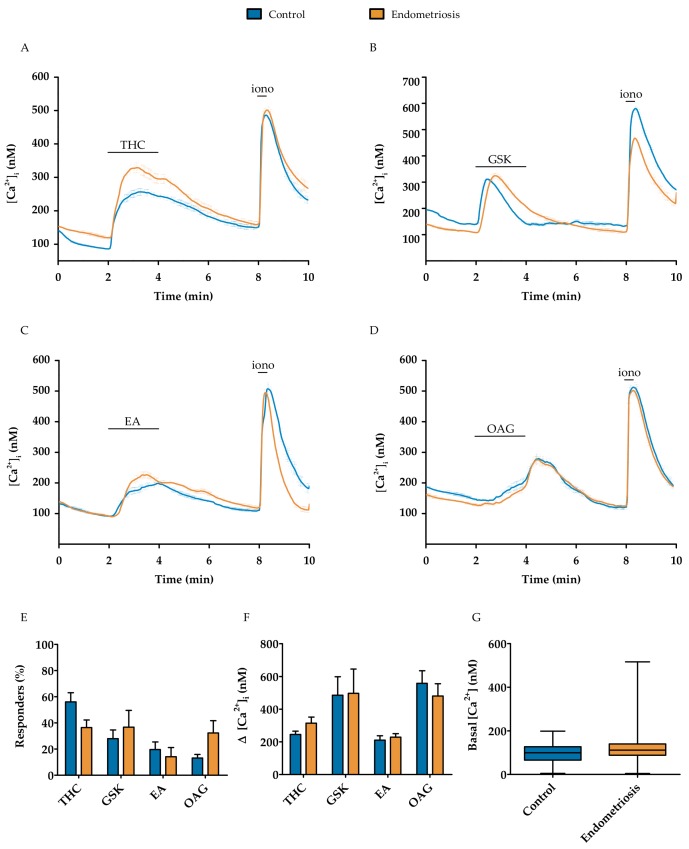
**The functional expression of TRPV2, TRPV4, TRPC1/4, and TRPC6** using Ca^2+^ microfluorimetry in hESC of endometriosis patients compared to the controls. (**A**–**D**) Average ± SEM traces of 50 µM THC-, 10 nM GSK-, 250 nM EA-, and 100 µM OAG-induced Ca^2+^ changes ([Ca^2+^]_i_), respectively, in control and endometriosis hESC; (**E**) Number of responders in which an increase of at least 100 nM of the intracellular calcium concentration was observed upon application of the agonist; (**F**) Increase in intracellular calcium upon application of TRP agonists; (**G**) Basal intracellular calcium concentration of responders. Data are presented as mean + SEM. Statistically significant changes in responders and calcium influx were assessed using the Two-way ANOVA statistical test with Bonferroni correction, basal calcium levels using a *t*-test.

TRPV2 functionality was investigated by the application of 50 µM THC, which elicited a robust calcium influx in both endometriosis-derived and control hESC, of 314 ± 73 nM in 36 ± 12% of all cells (total of 171 cells) and 244 ± 42 nM in 56 ± 14% of all cells (*n* = 314 cells) (Figure 3A,E,F and Figure S3A), respectively. GSK (10 nM) was used as a selective TRPV4 agonist, which showed a rapid and reversible calcium influx of 496 ± 366 nM in endometriosis-derived hESC and 484 ± 277 nM in control hESC, in, respectively, 37 ± 18% (total of 305 cells) and 28 + 16% of the cells (total of 241 cells) (Figure 3B,E,F and Figure S3B). The functional expression of TRPC1/4 was tested using 250 nM EA in the abovementioned protocol. Again, the basal calcium increased via a rapid and reversible calcium influx of 228 ± 47 nM in endometriosis-derived hESC and 210 ± 60 nM in control hESC, upon application of EA in, respectively, 14 ± 17% (total of 354 cells) and 20 ± 13% of the cells (total of 102 cells) (Figure 3C,E,F and Figure S3C). Analogously, 100 µM OAG was used to assess TRPC6 functionality, what resulted in an influx in intracellular calcium of 480 ± 198 nM in 32 ± 25% of the endometriosis-derived hESC responders (total of 114 cells). In control hESC, an increase of 557 ± 171 nM was observed in 13 ± 6% of the cells (total of 107 cells) (Figure 3D–F and Figure S3D). Overall, no significant differences were observed between hESC derived from controls or endometriosis patients when the percentage of responders, response amplitude, or basal calcium concentration were compared (Figure 3E–G). In conclusion, these results displayed no significant differences in the functional expression of TRP channels in primary hESC between control and endometriosis groups.

### 2.3. Proliferative and Migratory Capacity of Endometriosis-Derived hESC

**Figure 4:**
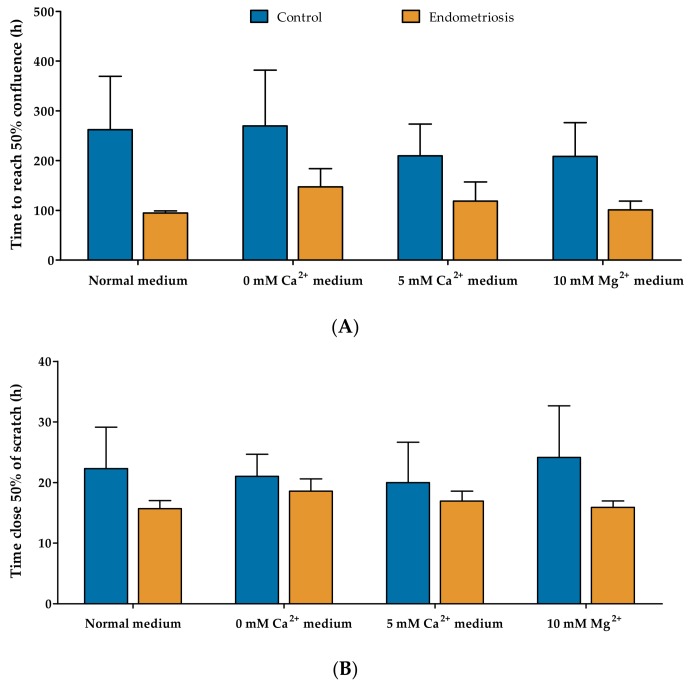
**Proliferative and migratory assay of endometriosis-derived hESC.** (**A**) The proliferation rate of hESC derived from eutopic endometrium of controls (*n* = 4) and endometriosis patients (*n* = 4) was assessed by calculating the time taken to reach 50% confluence in the presence of normal medium, 0 mM Ca^2+^, 5 mM Ca^2+^, and 10 mM Mg^2+^; (**B**) The migratory capacity of hESC derived from endometriosis patients (*n* = 4) compared to control hESC (*n* = 4). Using the scratch-wound assay, the time needed for 50% closure was calculated. Data are presented as mean + SEM. Statistically significant changes in proliferative and migratory rate were assessed using the Two-way ANOVA statistical test with Bonferroni correction.

TRP channel activity can elicit physiological reactions in organisms and cells by an influx of extracellular Ca^2+^ or by inducing changes in membrane potential. Moreover, a few of the TRP channels expressed in hESC are involved in the Ca^2+^ and Mg^2+^ homeostasis of the cell and regulate processes like cell migration, adhesion, and proliferation [27,28,29,30,31,32,33,34,35,36]. Thus, in light of endometriosis, the proliferative and migratory capacity of hESC was investigated in different Ca^2+^ and Mg^2+^ concentrations. Different media were used in order to further elucidate the contribution of TRP channels in these processes: normal medium, 0 mM Ca^2+^, 5 mM Ca^2+^, and 10 mM Mg^2+^. The mean time to reach 50% confluence was 262 ± 186 h, 269 ± 193 h, 209 ± 90 h, and 208 ± 96 h, respectively, for the control hESC; and 94 ± 5 h, 147 ± 63 h, 118 ± 54 h, and 100 ± 30 h for endometriosis-derived hESC (Figure 4A). No statistical differences were neither observed between the different cell types nor within the different conditioned media. A trend towards faster proliferation of the endometriosis-derived hESC could be observed in the normal culture medium, which should be noted (Two-way ANOVA column factor 0.03). Analogously, the migratory capacity was tested using the scratch-wound assay, resulting in 22 ± 11 h, 21 ± 6 h, 20 ± 11 h, and 24 ± 14 h for the control hESC, respectively; and 15 ± 2 h, 18 ± 3 h, 16 ± 2 h, and 15 ± 1 h for endometriosis-derived hESC to close 50% of the wound. Again, no statistical differences were observed neither between the different cell types nor within the different conditioned media. Overall, these results showed no differences in the proliferative and migratory capacity between control and endometriosis-derived eutopic stromal cells.

### 2.4. Eutopic vs. Ectopic

**Figure 5:**
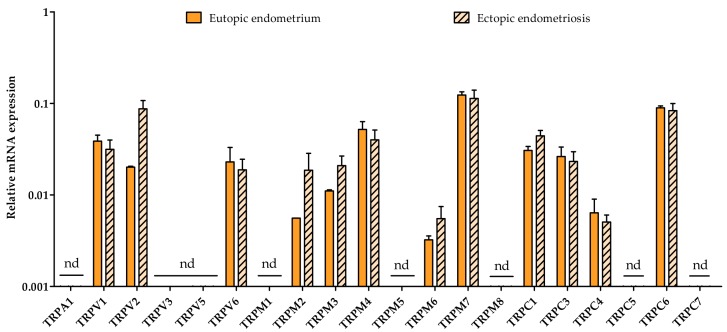
**TRP channel expression in eutopic versus ectopic endometrial biopsies**. RT-qPCR of TRP channels paired eutopic and ectopic tissue from grade II endometriosis patients during the follicular phase (*n* = 3) [37]. Messenger RNA levels were quantified to the geometric mean of housekeeping genes ACTB, GAPDH, HPRT, PGK-1, and TBP. TRPM1, TRPM5, TRPM8, TRPC5, and TRPC7 were below the detection limit. nd: not detectable. Data are presented as mean + SEM. Statistically significant changes in mRNA expression were assessed using the Two-way ANOVA statistical test with Bonferroni correction.

In order to elucidate whether the TRP expression pattern is different in ectopic lesions compared to their eutopic counterparts, RT-qPCR of all TRP channels was performed on paired tissue samples. The expression levels showed no significant difference between whole eutopic and ectopic tissue from endometriosis patients (Figure 5), which is also illustrated by a Spearman correlation coefficient of 0.95 (Figure S4). TRPV1, TRPV2, TRPV6, TRPM2, TRPM3, TRPM6, TRPM7, TRPC1, TRPC3, TRPC4, and TRPC6 were expressed well above the detection limit of the RT-qPCR analysis. Whereas TRPA1, TRPV3, and TRPV5 were observed to be expressed around the detection limit (30 < Cq < 35). TRPM1, TRPM5, TRPM8, and TRPC5 expression was not detected (Cq ≥ 35), which is similar to the findings in the endometrial biopsies.

## 3. Discussion

Although endometriosis is a highly prevalent disease, the etiology remains an enigma for researchers and clinicians. Laparoscopic surgery during the menstrual phase has shown that blood can be observed within the abdominal cavity, which makes the case of Sampson’s theory of retrograde menstruation a plausible etiological track. As this is a natural process, the endometrial debris that arrives in the abdominal cavity must obtain additional properties in order to thrive in this foreign location, i.e., have an improved ability to migrate, adhere, proliferate, and induce neuroangiogenesis. These processes, which require detailed signaling, use calcium as an indispensable second messenger [22,23]. In this context, members of the TRP superfamily have to be considered as possible contributors due to their role as cellular sensors [38], their functional expression in the endometrium [7], and their involvement in angiogenesis, proliferation, and migration [25,39,40]. In the present study, we assessed the expression pattern of TRP channels at the mRNA and functional levels, together with the proliferative and migratory capacity of the stromal cells in eutopic endometrium from endometriosis patients, in comparison with controls. Furthermore, the similarity of TRP channel expression was assessed between eutopic and ectopic tissue from endometriosis patients.

Using RT-qPCR, the expression profile of TRP channels was determined in whole endometrium biopsies throughout the menstrual cycle in tissue originating from endometriosis patients. TRPV1, TRPV2, TRPV4, TRPV6, TRPM4, TRPM6, TRPM7, TRPC1, TRPC3, TRPC4, and TRPC6 expression levels were observed well above the detection limit. For most TRP channels, fluctuations of the mRNA levels can be observed between the different menstrual phases. Especially for TRPM3 and TRPM6, significant differences between the menstrual-follicular-late luteal phase and the menstrual-early luteal phase, respectively, were measured. Unfortunately, the regulation of TRP channels by steroid sex hormones in the endometrium has only been studied for a limited amount of channels. For example, TRPV6 expression can be positively regulated by estrogen during the follicular phase, both in endometrial biopsies and Ishikawa cells [41]. TRPM2 mRNA expression has been investigated in human endometrium and hESC, revealing an increase in TRPM2 expression upon estrogen treatment [42]. The co-application of estrogen and progesterone results in an increase of TRPC1 mRNA, whereas TRPC6 expression increases solely by application of estrogen on hESC [43].

In the context of heart and kidney development in mice, in vivo administration of the steroid-derived cortisol to pregnant mice resulted in an increase of TRPM6 and TRPM7 expression in both organs [44]. As glucocorticoid levels are increased in rats when estrogen levels are high [45], the regulation of TRPM6 and TRPM7 in the endometrium might be caused by these steroid hormones. Unfortunately, consensus has not yet been reached regarding the glucocorticoid levels during the menstrual cycle in humans [46]. Regulation of TRPM3 by steroid hormones has only been studied on a functional level, showing TRPM3 inhibition by high progesterone levels in vitro [47]. Moreover, the fluctuation in TRP channel expression levels might not fully be attributed to the menstrual cycle. The local production of estrogen by the stromal cells, giving the disease an estrogen-dependent character, can also influence the regulation of TRP expression. In addition, samples were obtained from different patients with diverse reasons of fertility, which could explain the inter-sample variability.

The overall expression pattern of TRP channels throughout the menstrual cycle of endometriosis patients was synonymous with the earlier findings wherein controls were investigated [7]. In addition, these results are in line with previous reports in which endometrial tissue of control and endometriosis patients was investigated for potential biomarkers [48]. This meta-analysis did not show evidence for TRP channels as a meaningful biomarker to diagnose endometriosis, indicating that there are no significant differences in the RNA expression pattern.

Due to the involvement of TRP channels in processes such as migration, adhesion, and proliferation, the interest arose whether they contributed to the intrinsic characteristics of stromal cells, and hence also to the establishment of endometriosis. To this end, hESC cells were isolated from grade II endometriosis patients [37], which were collected during the luteal phase, as this results in the highest yield. Again, TRP channel expression was determined using RT-qPCR, although only the channels for which expression was present in the endometrial biopsies were taken into account. TRPV1, TRPV2, TRPV4, TRPM4, TRPM7, TRPC1, TRPC3, TRPC4, and TRPC6 mRNA was observed to be present in hESC of both endometriosis patients and controls. No expression of TRPM2 and TRPM3 was observed in both groups, although the former has been described to be expressed in hESC [42]. The ablation of TRPM2 and TRPM3 expression, however, can be explained by the loss of nerve endings when setting up the primary culture of hESC. The absence of TRPV6 and TRPM6 expression confirms the stromal identity of the cells, as these channels are known to be expressed in the epithelial cells [41,49]. Again, no significant differences could be observed between TRP channel expression in hESC derived from controls versus endometriosis patients.

Investigating TRP channel activity can be challenging at times, due to the limited number of selective agonists and antagonists. Therefore, only the functionality of TRPV2, TRPV4, TRPC1/4, and TRPC6 was investigated using Ca^2+^ microfluorimetry in this present study. Moreover, De Clercq et al. has already performed an elaborate screening of TRP channel activity in control hESC [7]. Thus, the present study merely aims to corroborate these findings in hESC derived from endometriosis patients. The functionality of TRPV2 in endometriosis-derived hESC was investigated by using the cannabinoid THC, which revealed a robust increase in intracellular Ca^2+^ concentration. This influx of calcium is similar to that observed in control hESC, and was elicited in a similar percentage of responders. These findings can also be extended to the functionality measurements of TRPV4 and TRPC6. In this study, we showed, for the first time, the functional expression of TRPC1/4, since stimulation by the selective agonist Englerin-A induced robust influxes in the intracellular Ca^2+^ concentrations. However, the EA-induced Ca^2+^ influxes were similar in both the control and endometriosis groups. Additionally, no difference could be observed in the basal Ca^2+^ concentration of the hESC derived from endometriosis patients, indicating no increased basal activity of endogenously expressed Ca^2+^-permeable TRP channels.

As proliferation and migration are important pathophysiological aspects of endometriosis, two assays were performed to elucidate whether TRP channels are involved in the establishment of the disease. Literature already showed the importance of TRPM4 in the proliferation of HeLa and PC3 cells, as TRPM4 silencing results in dropping-off proliferation rates of these cell lines, while overexpression caused a reciprocal effect [31,50]. TRPM7 has recently been linked to breast cancer cell proliferation [35]. This ion channel is not only a regulator of the Mg^2+^ homeostasis [36], but intracellular Mg^2+^ has been shown to regulate its activity. TRPC6 was shown to have an impact on the migration, sprouting, and proliferation of endothelial cells [39], while activation of TRPV1 stimulates migration of HepG2 cells [28]. Moreover, TRPC1 has been proposed to be involved in the cell migration process as a mechanosensor [51].

Our results showed that in standard cell culture conditions, hESC derived from endometriosis patients have a trend for improved cell proliferation, compared to control hESC. Similar observations were already made by Wingfield et al., who described increased cell proliferation in the endometrium of endometriosis patients via immunostaining scores [52]. However, since the expression of TRP channels in stromal cells was similar between endometriosis and control samples, this tendency for increased proliferation rates cannot be explained by a difference in TRP channel expression. As differences in TRP channel expression could express itself in the modified ability of hESC to thrive at extreme conditions (i.e., low or high levels of extracellular Ca^2+^ and Mg^2+^), hESC were subjected to these conditions, and their proliferation rate was measured subsequently. If the proliferation process of endometriosis-derived hESC is influenced by modulation of TRP channel expression, one would expect an altered proliferation rate when these cells are cultured in low Ca^2+^ medium compared to controls. Interestingly, no differences in proliferation rate were measured between control and endometriosis hESC, nor compared to the control situation. Similar reasoning processes could apply to extreme Ca^2+^ and Mg^2+^ concentrations in the extracellular medium—when TRP channel expression is altered, changes in proliferation rate could be expected when these cells are subjected to high calcium and magnesium levels. Overall, these findings might indicate that TRP channels do not play a vital role in the proliferation rate of hESC. Similar results were obtained when the migratory capacity was assessed. The scratch-wound assay indicated that endometriosis-derived hESC migrated at an equal rate, compared to control hESC. Furthermore, the depletion or supplementation of the media with Ca^2+^ and Mg^2+^ did not alter the migratory capacity of the cells. Since the expression pattern of TRP channels was similar between endometriosis and control samples, no differences in calcium and magnesium homeostasis were expected. Nevertheless, these results do not completely brush aside the involvement of TRP channels in proliferation and migration. The epithelial compartment and the endometrial stem/progenitor cells of the endometriotic lesions could still experience an increased proliferative and migratory capacity. For example, the epithelial TRPV6 has been studied extensively in the context of proliferation and cancer, showing increased metastasis and tissue invasion when prostate cancers are TRPV6-positive [53].

To address whether the ectopic environment can affect TRP channel expression in the endometrium, RT-qPCR was performed on paired eutopic biopsies and ectopic lesions from the follicular phase. Between these, no significant differences were found. Furthermore, these results are in line with our results on whole endometrial biopsies.

A first comment, however, is that the expression of TRP channels does not necessarily correlate with the functionality of the channel. The gating of the ion channel can be altered, even when no changes in expression are observed. Furthermore, channels could even lose their characteristic function and serve the cell in other ways, such as protein trapping. Therefore, a lack of alterations in TRP channel expression does not necessarily mean a lack of function for TRP channels, as some channels can employ a non-channel function [54]. Moreover, variations in passage numbers of hESC could also interfere with the observed expression of TRP channels [55]. Secondly, there is a lack of functional data of TRP channels expressed in the hESC of ectopic lesions. Unfortunately, isolation of primary cells from these lesions appears to be challenging and does not allow large yields, making functional measurements rather strenuous. Additionally, due to the scarcity of specific TRP pharmacology, not all expressed TRP channels in hESC could be examined on their functionality. Another remark is that excised lesions are located in a perimeter of healthy tissue. Although the lesions were cut out of this perimeter before RT-qPCR experiments were performed, not all surrounding tissue can be eliminated. Thus, the lesion comprises a heterogeneous pool of cells, i.e., peritoneal, mesothelial cells from surrounding tissue, nerve endings, endothelial cells, and stromal and epithelial cells. Finally, these results indicate no direct evidence for TRP channels in the development of the disease—however, it is possible that they are of great importance for the subfertility and pain symptoms that endometriosis patients experience in later stages of the disease.

In conclusion, our findings indicated that the endometrium of endometriosis patients and its stromal cells showed an unaltered expression pattern of TRP channels compared to controls. In addition, the proliferative and migratory capacity between the two groups was not significantly different, even in conditions where extracellular Ca^2+^ and Mg^2+^ levels were increased or reduced. Moreover, no differences in expression levels were observed between paired eutopic biopsies and ectopic lesions. Overall, this study suggests a similar TRP channel expression profile in the endometrium of endometriosis patients compared to controls, and provide strong evidence that dysregulation of TRP channel expression in hESC is not a major cause in the development of endometriosis.

## 4. Materials and Methods

### 4.1. Patients

Samples were obtained from women of reproductive age undergoing diagnostic laparoscopic surgery for pain and/or infertility at the Leuven University Fertility Centre (LUFC), UZ Leuven, Belgium, and who had received no hormonal therapy in the last 30 days. Women who were diagnosed with an American Fertility Society score grade 0 were considered as controls, whereas those of grade II or more were considered as patients with endometriosis [37]. The use of endometrial and endometriotic tissue was approved by the Institutional Ethical and Review Board of the University Hospital of Gasthuisberg for the protection of human subjects (ML9100-S54776 date 04/10/2012). Written informed consent was obtained from all participating subjects.

### 4.2. Samples

Methods were based on established protocols from our research group [7].

#### 4.2.1. Whole Endometrium Biopsies from Endometriosis Patients for TRP Expression Studies throughout the Menstrual Cycle

Endometrial biopsies were selected from the biobank of the LUFC. Detailed patient information is provided in Table S1, including the endometriosis grade, AFS score, BMI, age, endometriosis score, and reason for infertility. These samples were obtained using a sterile Novak-curette in the operating room after hysteroscopy and before laparoscopy. The stage of the menstrual cycle was determined from the patient’s menstrual history and confirmed by endometrial histology, according to the Noyes criteria [56]. Endometriosis grade II endometrial samples were obtained from the menstrual (days 1–5, *n* = 5), follicular (days 6–14, *n* = 6), the early luteal (days 15–20, *n* = 4), and the late luteal phase (days 21–28, *n* = 3) (Table S1). All samples were collected between 2002 and 2014, snap frozen in liquid nitrogen, and stored in the LUFC biobank at −80 °C until used.

#### 4.2.2. Primary Human Endometrial Stromal Cells

Cultures of primary human endometrial stromal cells (hESC) were started from fresh endometrial biopsies (*n* = 16), obtained from women during the luteal phase, of both endometriosis grade II and III patients and controls (Table S2). After rinsing the biopsy with phosphate-buffered saline (PBS), mucus and blood were removed with scalpels. The tissue was manually minced into pieces smaller than 1 mm^2^ and incubated with 0.2% collagenase type IA (Sigma-Aldrich, Bornem, Belgium) in Dulbecco’s modified Eagle’s Medium (DMEM) (Gibco, Invitrogen, Ghent, Belgium) for 60 min at 37 °C with constant shaking. The hESC and human endometrial epithelial cells were separated by differential size using gravity sedimentation, as described previously [57]. The hESC were cultured in DMEM/F-12 containing 10% fetal bovine serum (FBS, Gibco), 0.5 µg/mL amphotericin B (Gibco), and 100 µg/mL gentamicin (Gibco), and kept at 37 °C in a humidified, 5% CO_2_, 95% air atmosphere [58]. Cells were routinely sub-cultured when they reached 80–90% confluence. The medium was changed every 2–3 days. To assess the expression profile of TRP channels in hESC, cells were frozen, stored at −80 °C and used RT-qPCR. Note that hESC from different patients and different passages (2–9) were used.

#### 4.2.3. Whole Endometrium Biopsies and Endometriosis Lesions from Endometriosis Patients for Paired TRP Expression Study

Endometrial biopsies were obtained using a sterile Novak-curette in the operating room after hysteroscopy and before laparoscopy. The stage of the menstrual cycle was determined from the patient’s menstrual history and confirmed by endometrial histology, according to the Noyes criteria [56]. Endometriosis lesions were taken during the laparoscopy using a CO_2_-laser. Endometriosis grade II samples were obtained from the follicular phase (days 6–14, *n* = 3), which were classified as superficial lesions on the peritoneum (Table S3). Both endometrial biopsies and endometriotic lesions were left for 48 h on the RNALater (Qiagen, Venlo, The Netherlands) and stored at −80 °C until further RT-qPCR analysis.

### 4.3. RT–qPCR Experiments

Methods were based on established protocols from our research group [7].

#### 4.3.1. Whole Endometrial Biopsies and Endometriotic Lesions

RT–qPCR experiments were performed on RNA isolated from frozen whole endometrial biopsies (*n* = 21) and endometriotic lesions (*n* = 3). The tissue was homogenized by use of a power homogenizer (Polytron, Montreal, QC, Canada) and total RNA was extracted with TriPure Isolation Reagent (Roche, Mannheim, Germany). RNA concentrations were assessed using the Nanodrop method (Isogen Life Science, Temse, Belgium) and RNA quality was assessed using an Experion RNA StdSens Analysing kit (Bio-Rad, Nazareth Eke, Belgium) (good quality RNA samples included an RNA integrity number 7 for all samples). 1 µg RNA was subsequently used for cDNA synthesis using the High-Capacity cDNA Reverse Transcription Kit (Life Technologies Europe B.V., Ghent, Belgium), and Triplicate cDNA (2.5 × diluted) samples from each independent preparation were used in the StepOne PCR system (Applied Biosystems, Life Technology, Carlsbad, CA, USA) using specific TaqMan gene expression assays for all TRP channels (Table S4). For each gene, 2 µL cDNA was added to 5 µL Mastermix (Life Technology), as well as 2.5 µL diethylpyrocarbonate-treated RNase-free water and 0.5 µL of the specific TaqMan gene primer, resulting in a final volume of 10 µL. ACTB, GAPDH, HPRT1, PGK-1, and TBP were used as endogenous controls. The protocol consisted of a holding stage at 95 °C for 20 min followed by a cycling stage of 40 replication cycles at 60 °C for 20 min (StepOne PCR system, Applied Biosystems, Life Technology). TRP channels with Cq values above 35 cycles were considered as non-detectable. Data were shown as 2^(−ΔCq)^ (mean + SEM) in which ΔCq = Cq_TRP channel_ − Cq_geometric mean of endogenous controls_. Statistical tests were performed on the ΔCq values.

#### 4.3.2. Primary Human Endometrial Stromal Cells

hESC were obtained as described above. RT–qPCR experiments were performed on RNA isolated from human endometrial stromal cells derived from endometriosis patients (*n* = 4) and controls (*n* = 3) (cell passage 1 or 2). Total RNA was extracted with the RNeasy mini kit (Qiagen, Venlo, The Netherlands). RNA concentrations were assessed using the Nanodrop method (Isogen Life Science, Temse, Belgium) and quality of the RNA was assessed using an Experion RNA StdSens Analysing kit (Bio-Rad, Nazareth Eke, Belgium) (good quality RNA samples included an RNA integrity number 7 for all samples). 1 µg RNA was subsequently used for cDNA synthesis using the First-Strand cDNA Synthesis Kit (GE Healthcare, Chicago, IL, USA). Triplicate cDNA (2.5 × diluted) samples from each independent preparation were used in the StepOne PCR system (Applied Biosystems, Life Technology) using specific TaqMan gene expression assays for all TRP channels (Table S4). For each gene, 2 µL cDNA was added to 5 µL Mastermix (Life Technology), 2.5 µL diethylpyrocarbonate-treated RNase-free water, and 0.5 µL of the specific TaqMan gene primer, resulting in a final volume of 10 µL. The MS Excel application GeNorm 3.5 indicated PGK-1 and HPRT1 as the most stable endogenous controls for further analysis. The protocol consisted of a holding stage at 95 °C for 20 min, followed by a cycling stage of 40 replication cycles at 60 °C for 20 min (StepOne PCR system, Applied Biosystems, Life Technology). TRP channels with Cq values above 35 cycles were considered as non-detectable. Data were shown as 2^(−ΔCq)^ (mean + SEM) in which ΔCq = Cq_TRP channel_ − Cq_geometric mean of endogenous controls_. Statistical tests were performed on the ΔCq values.

### 4.4. Functional Measurements

The protocol and imaging system for standard Ca^2+^-measurements were performed as described by Vriens et al. [59]. As selective pharmacological agents for TRP channels are limited, not all TRP channels have been tested for their functional expression. The functionality of TRPV2, TRPV4, TRPC1/4, and TRPC6 was assessed by the application of 50 µM *∆9*-tetrahydrocannabinol (THC) [60], 10 nM GSK016790A (GSK) [61], 250 nM Englerin A (EA) [62], and 100 µM 1-oleoyl-2-acetyl-glycerol (OAG) [63], respectively. 2 μM ionomycin (iono; Sigma) was applied at the end of every experiment as a positive control.

For intracellular Ca^2+^ measurements, hESC were incubated with 2 μM Fura-2 acetoxymethyl ester (Invitrogen, Eugene, OR, USA) for 30 min at 37 °C. Fluorescent signals were evoked during alternating illuminations at 340 and 380 nm using a Lambda XL illuminator (Sutter instruments, Novato, CA, USA), and recorded using an Orca Flash 4.0 camera (Hamamatsu Photonics Belgium, Mont-Saint-Guibert, Belgium) on a Nikon Eclipse Ti fluorescence microscope (Nikon Benelux, Brussels, Belgium). The imaging data were recorded and analyzed using the NIS-elements software (Nikon). Absolute calcium concentrations were calculated from the ratio of the fluorescence signals at both wavelengths (F340/F380) after correction for the individual background fluorescence signals, using the Grynkiewicz equation [64]. The standard solution contained 150 mM NaCl, 2 mM CaCl_2_, 1 mM MgCl_2_, 10 mM d-glucose, and 10 mM HEPES (pH 7.4 with NaOH). Note that the cell passages ranged from 2 to 9.

For all measurements, cells were considered responders if the calcium influx during agonist application exceeded 100 nM and when the highest value of the derivative of the calcium trace during the application of an activator exceeded at least 3 times the standard deviation of the derivative during basal conditions. Calcium amplitudes were calculated as the difference between the maximum Ca^2+^ and basal Ca^2+^ of responding cells during the application of an activator. Only cells that responded to ionomycin at the end of the experiment were considered.

### 4.5. Proliferation Assay

hESC cell proliferation was evaluated by collecting real-time data of cell confluence using the IncuCyte^®^ ZOOM Live-Cell Analysis System (Essen BiosScience, Ann Arbor, MI, USA). hESC were seeded using a normal growth medium containing 2.9 mM Ca^2+^ and 3.2 mM Mg^2+^ (as described above) into 24-well culture plates (Greiner Bio One, Belgium) at a cell density of 10,000 cells/well. Cells were left to attach for 30–45 min before the medium was replaced to obtain the control, calcium-free, 5 mM calcium, and 10 mM magnesium culture conditions (as described above). hESC were placed into the IncuCyte^®^ ZOOM Live-Cell Analysis System and live images were taken every 2 h over a period of 5 days. Cell proliferation data was obtained via the cell confluence increment in each of the conditions, after which the time taken to reach 50% confluence was calculated. Note that the cell passages ranged from 2 to 9.

### 4.6. Migration Assay

The migration potential of hESC derived from endometriosis patients was assessed via the scratch assay [65]. Isolated hESC were seeded into 12-well culture plates and grown to a monolayer in normal growth medium containing 2.9 mM Ca^2+^ and 3.2 mM Mg^2+^ (as described above). When cells reached 100% confluency, the medium was removed and the monolayer was scraped in the middle of the well to create a ‘scratch’ using a p200 pipet tip. Cells were gently washed with PBS to remove all debris and to smoothen the edges of the scratch, and medium was added onto the wells. Standard growth medium (as described above) was used as the control condition, and was supplemented with 1 mM ethylene glycol-bis(β-aminoethyl ether)-*N*,*N*,*N*′,*N*′-tetraacetic acid (EGTA), 4 mM Ca^2+^ or 9.7 mM magnesium, to obtain the calcium-free, 5 mM calcium, or 10 mM magnesium culture conditions, respectively. Markings were made onto the culture wells as reference points to obtain the same field during image acquisition. Images were taken at the time of scratching, and at least 3 different time points thereafter using a Nikon Eclipse Ts2 microscope. In between image acquisition, cells were kept at 37 °C in a humidified 5% CO_2_ incubator. At the end of the experiment, images were analyzed using Image J, Fiji Software (National Institute of Health, USA) [66], and the time taken to reach 50% closure of the scratch wound was determined. Note that the cell passages ranged from 2 to 9.

### 4.7. Data Analysis and Display

For data display, the Origin 9.0 Software package (OriginLab, Northampton, MA, USA) and Graphpad Prism 5.01 (Graphpad software incorporated, La Jolla, CA, USA) were used. The latter was also implemented for statistical analysis.

## Supplementary Materials

Supplementary materials can be found at http://www.mdpi.com/1422-0067/19/9/2467/s1.
