# Supplementary material for: Functional Expression of TRP Ion Channels in Endometrial Stromal Cells of Endometriosis Patients
Source: Int J Mol Sci. 2018 Aug 21;19(9):2467. doi: 10.3390/ijms19092467 (PMC6163224; doi:10.3390/ijms19092467)
Supplement: Supplementary file 1 [file ijms-19-02467-s001.pdf]

International Journal of *Molecular Sciences*
MDPI

5. Supplementary material

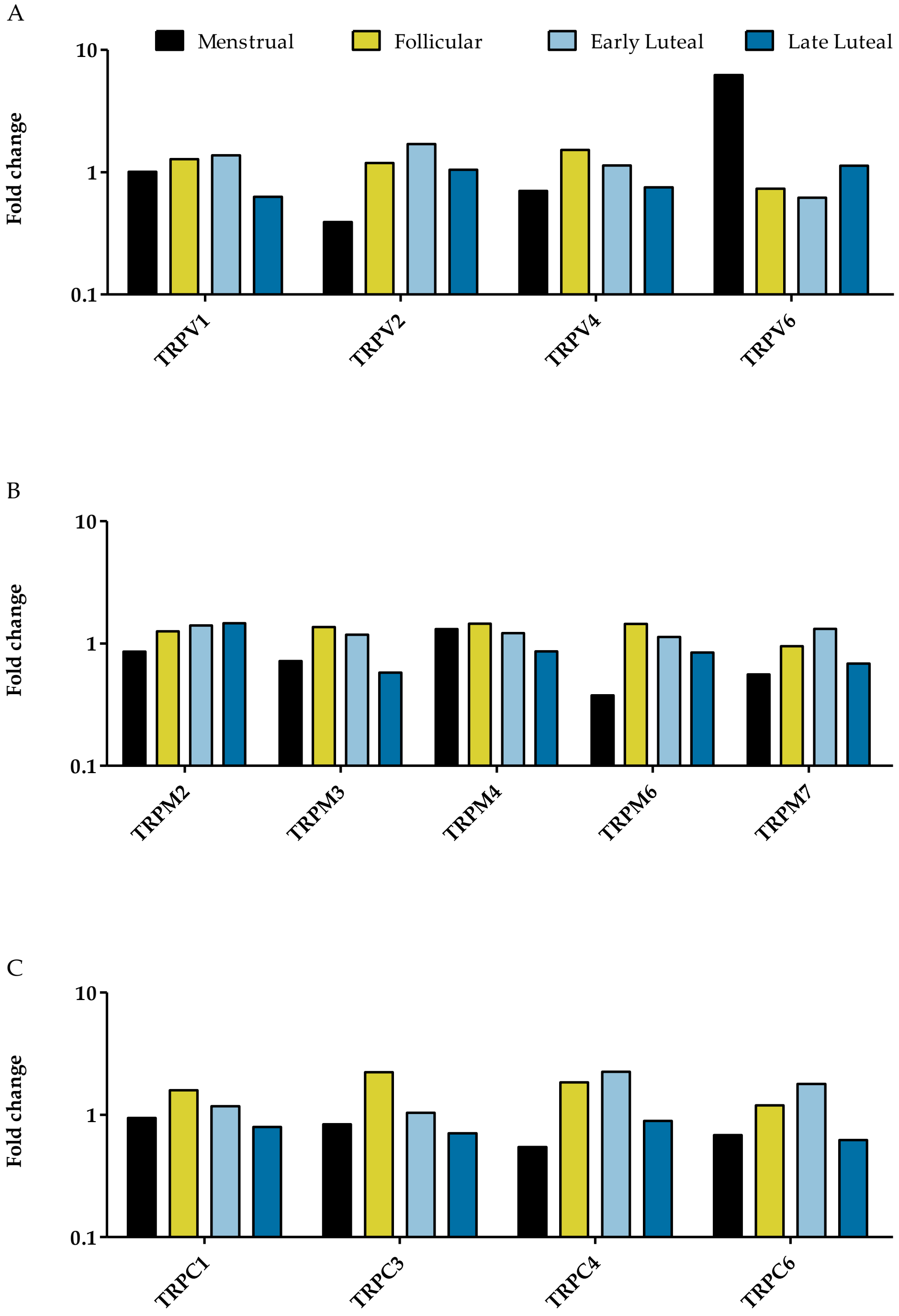

Figure S1. The fold change of mRNA expression to controls [7] **(a)** TRPV-A; **(b)** TRPM; **(c)** TRPC. Data shown as $2^{(-\Delta\Delta Cq)}$. ($\Delta\Delta Cq = \Delta Cq_{endometriosis} - \Delta Cq_{control}$)

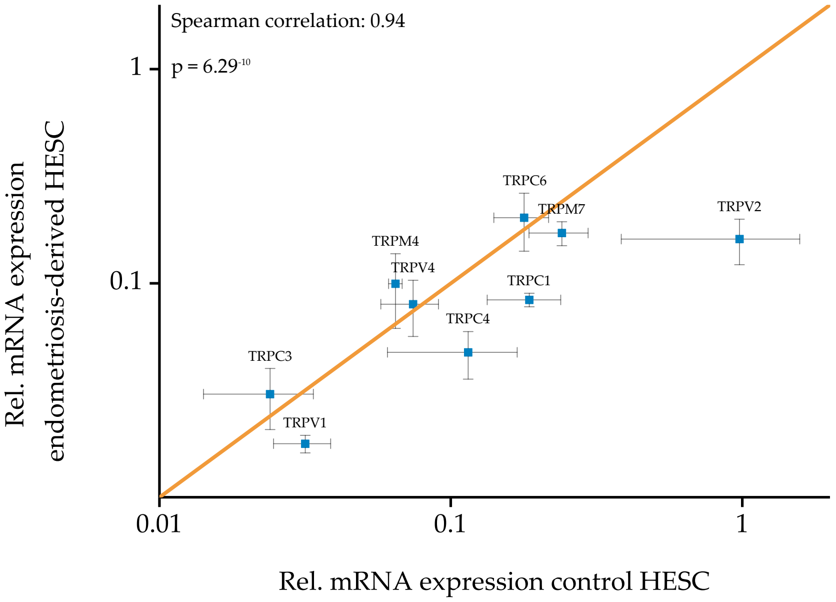

Figure S2. Correlation between the mRNA expression of TRP channels in control hESC (n = 3) and endometriosis-derived hESC (n = 4). The correlation was assessed using the non-parametric Spearman correlation test.

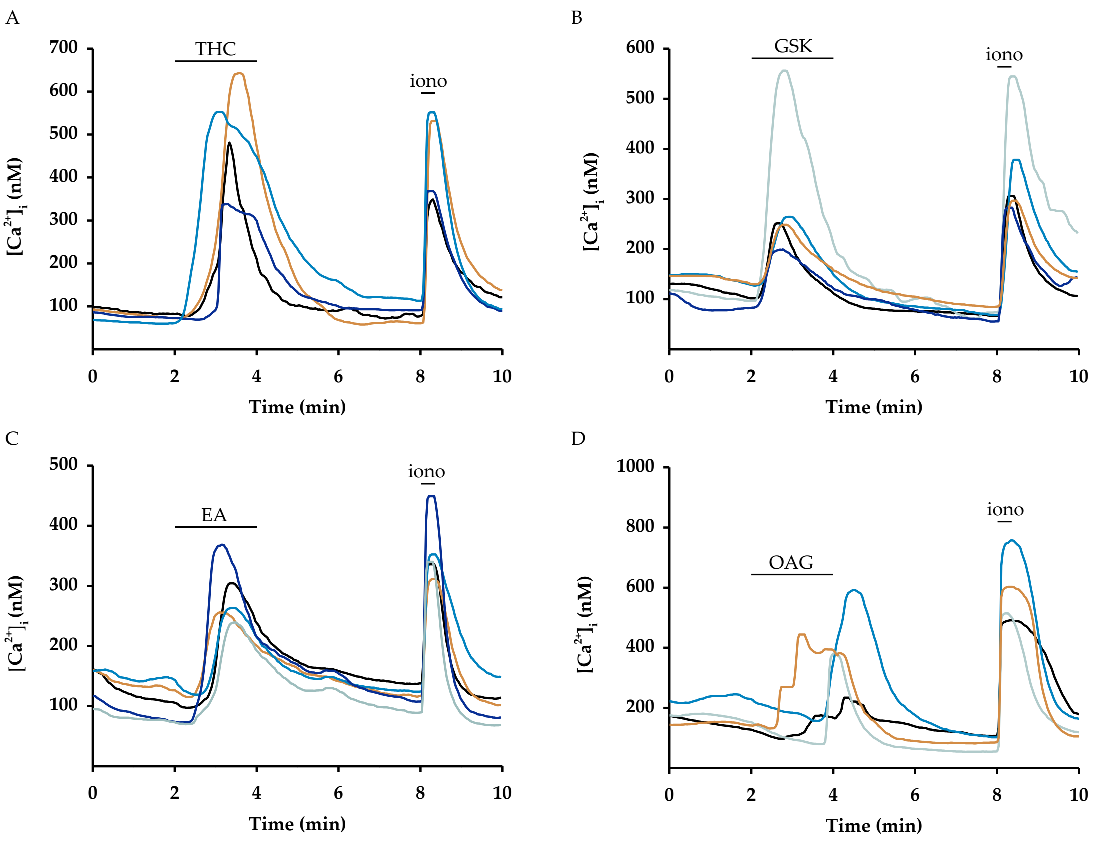

Figure S3. Representative traces of individual endometriosis-derived hESC illustrating TRP functionality (a-d) Application of respectively 50 µM THC, 10 nM GSK, 250 nM EA and 100 µM OAG elicit a robust and reversible influx of calcium in hESC derived from endometriosis-patients.

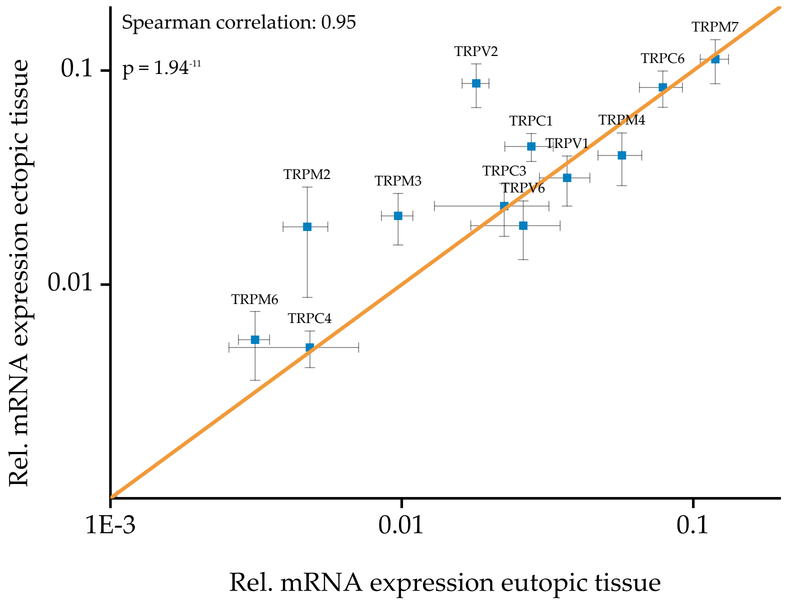

Figure S4. Correlation between the mRNA expression of TRP channels in paired eutopic and ectopic tissue samples (n=3). The correlation was assessed using the non-parametric Spearman correlation test.

International Journal of
Molecular Sciences

MDPI

ENDOMETRIAL BIOPSIES							
**Sample**	**Endometriosis stadium**	**AFS score**	**Cycle phase**	**Age (years)**	**BMI (kg/m²)**	**Smoking**	**Time interval between menses**
**1**	II	10	Menstrual	38	23.7	No	Normal
**2**	II	14	Menstrual	37	23.3	No	Normal
**3**	II	8	Menstrual	31	16.2	No	Normal
**4**	II	12	Menstrual	28	18.9	Yes	Normal
**5**	II	15	Menstrual	35	26.6	No	Normal
**1**	II	12	Follicular	32	21.8	No	Normal
**2**	II	10	Follicular	34	26.6	No	Normal
**3**	II	6	Follicular	21	19	Yes	Normal
**4**	II	10	Follicular	33	21.6	No	Normal
**5**	II	6	Follicular	29	21.9	No	Normal
**6**	II	8	Follicular	34	25.3	No	Normal
**1**	II	12	Early luteal	37	nd	nd	nd
**2**	II	18	Early luteal	33	20.8	No	Normal
**3**	II	10	Early luteal	30	29.8	No	Normal
**4**	II	6	Early luteal	35	19.6	No	Normal
**1**	II	8	Late luteal	31	20.4	No	Normal
**2**	II	8	Late luteal	26	20.5	Yes	Normal
**3**	II	10	Late luteal	31	17.6	No	Normal

Table S1. Demographics and information of cycle phases/days for each endometrial biopsy. Normal time between menses equals 24–38 days; nd: not determined due to lack of information**.**

PRIMARY HUMAN ENDOMETRIAL STROMAL CELLS												
**Sample**	**Endometriosis stadium**	**AFS score**	**Cycle phase**	**Age (years)**	**BMI (kg/m²)**	**Smoking**	**Time interval between menses**	**Experimental destination**			
								**qPCR**	**Ca^{2+}**	**Prol.**	**Migr.**
1	0	n/a	Luteal	29	19.2	Occasionally	Normal		X		
2	0	n/a	Luteal	31	21.2	No	Normal		X		
3	0	n/a	Luteal	26	31.6	Yes	Normal	X	X		
4	0	n/a	Luteal	40	18.6	No	Normal	X	X	X	X
5	0	n/a	Luteal	27	25.3	Yes	Infrequent	X	X	X	X
6	0	n/a	Luteal	29	24.6	No	Normal		X	X	X
7	0	n/a	Luteal	39	28	No	Normal		X	X	X
1	II	8	Luteal	31	20.2	No	Normal		X	X	X
2	II	12	Luteal	28	23	No	Normal	X	X		
3	II	8	Luteal	36	19.1	No	Normal		X	X	X
4	II	8	Luteal	30	20.8	No	Normal	X	X	X	X
5	II	8	Luteal	37	31.6	No	Normal	X	X		
6	II	8	Luteal	28	21.5	No	Normal	X	X	X	X
7	III	16	Luteal	34	24.5	No	Normal		X		
8	III	40	Luteal	27	19.8	No	Normal		X		

Table S2. Demographics and reproductive information for each endometrial biopsy. Normal time between menses equals 24–38 days; n/a: not applicable.

PAIRED EUTOPIC AND ECTOPIC TISSUE								
**Sample**	**Endometriosis stadium**	**AFS score**	**Cycle phase**	**Age (years)**	**BMI (kg/m²)**	**Smoking**	**Time interval between menses**	**Ectopic lesion**
1	II	8	Follicular	30	23.6	No	Normal	Superficial lesion; pararectal
2	II	8	Follicular	25	29.4	No	Normal	Superficial lesion; pararectal
3	II	10	Follicular	36	21.5	No	Normal	Superficial lesion; uterovesicular

Table S3. Demographics and reproductive information of the paired eutopic and ectopic tissues. Normal time between menses equals 24–38 days

**TAQMAN GENE EXPRESSION ASSAYS**					
**Gene name**	**Assay ID**	**RefSeq ID**	**Exon boundary**	**Assay location**	**Amplicon length**
TRPA1	Hs00175798_m1	NM_007332.2	2–3	441	124
TRPV1	Hs00218912_m1	NM_018727.5	8–9	1540	94
TRPV2	Hs00901640_m1	NM_016113.4	14–15	2591	72
TRPV3	Hs01000530_m1	NM_001258205.1	14–15	2128	121
TRPV4	Hs01099348_m1	NM_001177428.1	2–3	586	65
TRPV5	Hs00219765_m1	NM_019841.4	7–8	1176	96
TRPV6	Hs01114089_g1	NM_018646.4	7–8	1138	59
TRPM1	Hs00931865_m1	NM_001252020.1	2–3	448	91
TRPM2	Hs01066071_m1	NM_003307.3	9–10	1419	74
TRPM3	Hs00257553_m1	NM_001007470.1	3–4	356	83
TRPM4	Hs00214167_m1	NM_001195227.1	12–13	1852	60
TRPM5	Hs00175822_m1	NM_014555.3	18–19	1795	79
TRPM6	Hs01019353_m1	NM_001177310.1	13–14	1555	68
TRPM7	Hs00918956_m1	NM_017672.4	38–39	5745	148
TRPM8	Hs00375481_m1	NM_024080.4	2–3	157	106
TRPC1	Hs00608195_m1	NM_001251845.1	6–7	1097	137
TRPC3	Hs00162985_m1	NM_001130698.1	4–5	1416	58
TRPC4	Hs00211805_m1	NM_001135955.1	8–9	2312	76
TRPC5	Hs00202960_m1	NM_012471.2	6–7	2623	75
TRPC6	Hs00989190_m1	NM_004621.5	3–4	1558	87
TRPC7	Hs00220638_m1	NM_001167576.1	4–5	1458	55
ACTB	Hs01060665_g1	NM_001101.3	2–3	208	63
GAPDH	Hs02758991_g1	NM_001256799.2	6–7	752	93
HPRT1	Hs02800695_m1	NM_000194.2	2–3	297	82
PGK-1	Hs00943178_g1	NM_000291.2	5–6	697	73
TPB	Hs00427620_m1	NM_001172085.1	2–3	578	91

Table S4. List of used Taqman genes (Applied Biosystems).
